# Inhibitory-like Substances Produced by Yeasts Isolated from Andean Blueberries: Prospective Food Antimicrobials

**DOI:** 10.3390/foods12132435

**Published:** 2023-06-21

**Authors:** Gabriela N. Tenea, Blanca Anrango Cajas, Bladimir Carlosama Sanchez

**Affiliations:** Biofood and Nutraceutics Research and Development Group (GIDIBAN), Faculty of Engineering in Agricultural and Environmental Sciences, Universidad Técnica del Norte, Av. 17 de Julio s-21, Barrio El Olivo, Ibarra 100150, Ecuador

**Keywords:** yeasts, antimicrobial activity, pathogens, sucrose, EDTA, heat-induced antimicrobial action

## Abstract

Natural agents from microorganisms have emerged as suitable options to replace chemical preservatives in foods. In this study, the antibacterial activity of cell-free supernatant (CFS) from five native yeasts (*Saccharomyces cerevisiae* Lev6 and Lev30, *C. pseudointermedia* Lev8, *Candida intermedia* Lev9, *C. parapsilosis* Lev15) and the reference *S. boulardi* SSB, was evaluated against some indicator food pathogens. The generation of antimicrobials was reliant on strain-, and sugar-supplemented media, which supported yeast growth established at 30 °C and 200 rpm for 48 h. Treatment with proteinase K and catalase was unable to completely abolish the inhibitory effect, indicating that the active components are likely complex combinations of acids, proteins, hydrogen peroxide, and other metabolites. Although there was no impact on *Listeria monocytogenes*, exposure to CFS and extracellular fractions obtained through precipitation with methanol (PPm) at 120 °C for 60 min significantly (*p* < 0.05) increased the inhibitory activity against *Escherichia coli*, *Salmonella enterica*, *Kosakonia cowanii*, and *Staphylococcus aureus*, indicating that the inhibitory activity was stimulated by heat. Likewise, a synergistic inhibitory action against *Listeria* was obtained following the pretreatment of PPm with EDTA (ethylenediaminetetraacetic acid). These activities were yeast strain-dependent, with Lev6, Lev8, and Lev30 showing the highest activity. In addition, a heat-stable low-molecular-mass molecule under 5 kDa was detected in Lev30. Further research is required to evaluate the mode of action and characterize the composition of the released molecules in the CFS in order to develop a novel biocontrol agent based on yeasts.

## 1. Introduction

Foodborne diseases are caused by the ingestion of foods that contain harmful microorganisms (e.g., bacteria, fungi, viruses) capable of jeopardizing their quality [1]. If present in high concentrations, these microorganisms can also compromise health. Growing customer concerns about toxicity and antimicrobial-resistant pathogens in food have led to a search for alternative food preservation methods to assure microbiological quality and control food stability systems. Many chemicals have been extensively used to guarantee microbiological food safety and extend shelf life [2]. Unfortunately, these antimicrobials have led to several issues, including increases in antibiotic-resistant bacteria and environmental pollution, as well as greater risks to human health due to chemical residues [3]. Thus, research trends are focused on searching for naturally derived compounds that can replace food chemicals to address consumer demands for safety and security [4]. These molecules are typically derived from bacteria, plants, fermented foods, and other sources [3]. Many studies have examined the development of antimicrobials derived from lactic acid bacteria (LAB); however, few have concentrated on the generation of antimicrobials obtained from yeasts [5,6,7]. Yeasts are microorganisms that are found in various natural sources [8]. For example, due to its superior fermentation capabilities, *S. cerevisiae*, one of the most studied microbes, has long been used in various biotechnological applications. In addition to the industrial uses of yeasts, probiotics and health benefits have also been identified [9,10,11], as well as their potential to inhibit the growth of harmful food pathogens [12]. It has been demonstrated that the peptide isolated from *S. boulardii* has strong inhibitory activity against *Bacillus cereus* [5]. In addition, yeasts produce extracellular enzymes (i.e., proteases), toxins, enzyme-based proteins, organic acids, antibiotics, volatile acids, hydrogen peroxide, and free phenolics, among other antioxidant molecules with antibacterial properties [13,14,15]. The supernatant and lysate of *S. cerevisiae* cultures exert an anti-biofilm effect against *S. aureus* [16]. In addition, cell-free supernatant inhibits *B. subtilis*, *B. cereus*, *E. coli*, *Proteus vulgaris*, *Pseudomonas aeruginosa*, *S. typhimurium*, and *Yersinia enterocolitica* [8]. This collection of substances explains the dynamics and predominance of yeast cells in complex microbiomes, such as those found in spontaneous fermentations [17]. Other studies indicated that species of the *Candida* genus, an opportunistic group of yeasts, displayed inhibitory activity against putrefying fungi during fruit storage [18,19]. For example, *C. intermedia* was found to produce antifungal substances against the wine-spoilage strain *Brettanomyces bruxellensis* [6]. Likewise, *C. parapsilosis* produces biosurfactants such as sophorolipids used in cosmetics and therapeutics [20]. In addition, some yeasts release killer toxins with antibacterial properties [21,22].

In recent research, we showed the potential of some yeasts isolated from wild black Andean blueberries from Ecuador to produce ethanol from sugarcane juice [23], which increased the scientific interest in the exploration of their biotechnological properties, including the evaluation of the antimicrobial capacity of substances released in the supernatant during fermentation.

Considering the potential of yeasts in the food industry, the present study aims to evaluate the antimicrobial activity of some native yeasts against food pathogens to select suitable candidates that can be further used as natural preservatives. The effect of medium composition on the production of antimicrobial substances, as well as the inhibitory spectrum against some non-pathogenic and pathogenic microorganisms, was assessed. Moreover, the nature of these antimicrobials was evaluated in vitro, along with their sensitivity to pH, heat, inorganic, and organic treatments. Furthermore, the antimicrobial activity of concentrated and partially ultrapurified fractions was evaluated, and the molecular weight was estimated.

## 2. Materials and Methods

### 2.1. Yeast Strains

The yeast strains Lev6 (NCBI accession no. MZ768866), Lev9 (NCBI accession no. MZ768867), and Lev30 (NCBI accession no. MZ768868), which were previously isolated from a pool of exocarp and mesocarp of wild black dark-purple Andean blueberries (*Vaccinium floribundum* Kunth.) from the El Cristal subtropical rainforest (Santo Domingo de Los Tsáchilas), Esmeraldas Province, Ecuador, were used [23]. Similarly, two isolates, with the codes Lev8 and Lev15, were obtained from pale-green blueberries. Using 16S ARN sequencing, these isolates were classified as *C. pseudointermedia* (NCBI accession no. OP603448) and *C. parapsilosis* (NCBI accession no. OP603449). Stocks of these strains were maintained at −80 °C in 20% glycerol (*v*/*v*). Fresh cultures were obtained by cultivation on Yeast Peptone Dextrose (YPD) agar medium (Difco, Detroit, MI, USA) before use. As a reference for all experiments, the probiotic *S. boulardii* CNCM 1-745 (SSB) was used.

### 2.2. Antimicrobial Activity Assay

One mL of a 24-h-old yeast cell suspension culture was inoculated into 100 mL of YPD broth (M9) medium for 48 h at 30 °C with constant agitation at 200 rpm, followed by centrifugation for 20 min at 13,000× *g* at 4 °C. The CFS was recuperated and double-filtered using 0.45 μm and 0.22 μm porosity syringe filters (Chemlab Group, Washington, DC, USA). The pH was measured at the end of growth and it was stored at 4 °C until further use in the agar diffusion method, as described in [9]. For the antimicrobial activity evaluation, *E. coli* ATCC25922 was used as an indicator strain. Briefly, *E. coli* (1 × 10^7^ CFU/mL) was mixed with 3.5 mL of soft YPD agar (0.75%), swabbed onto nutrient agar plates, and incubated at 37 °C for 2 h. The CFS (100 μL) of each tested yeast was transferred into wells (6.0–6.5 mm) with overlay agar, incubated at 37 °C, and subsequently examined for inhibition zones at 48 h. The experiments were run in triplicate, and the mean value of the inhibition zone was determined using an automatic plate scanner (Scan500, Interscience, Fr, Saint-Nom-la-Bretèche, France). M9 medium was used as a negative control.

### 2.3. Effect of Medium Composition on Antimicrobial Activity

The basal M9 medium was modified as follows: (1) M1 consisted of M9 supplemented with 5% sucrose; (2) M2 consisted of M9 supplemented with 10% sucrose; (3) M3 consisted of M9 supplemented with 20% sucrose; (4) M4 consisted of M9 supplemented with 40% sucrose; (5) M5 consisted of M9 supplemented with 5% dextrose; (6) M6 consisted of M9 supplemented with 10% dextrose; (7) M7 consisted of M9 supplemented with 20% dextrose; (8) M8 consisted of M9 supplemented with 40% dextrose. The pH was adjusted to 6.0 before inoculation with the target yeasts. One ml of a 24-h-old yeast cell suspension culture was inoculated into each medium for 48 h and the CFS was further used in the agar well diffusion assay, as described in Section 2.2. The experiments were run in triplicate and the mean value of the inhibition zone was determined. M9 was used as a control. The residual inhibitory activity (RIA) (%) was calculated using the following equation: RIA (%) = 1 − (Ac − As/Ac) × 100, where Ac is the diameter of the inhibition zone of the control sample and As is the diameter of the inhibition zone of the test sample [7].

### 2.4. Yeast-Growth Kinetics and Production of Antimicrobial Substances

One ml of a 24-h-old yeast cell suspension culture (1 × 10^7^ CFU/mL) was inoculated into M2 (150 mL), as described in Section 2.3. The culture was then incubated for 48 h at 30 °C with agitation at 200 rpm. The optical density (OD605 nm) and pH were monitored at intervals of 3 h for 48 h. The production of antimicrobial compounds was also monitored at the same intervals during growth by measuring the inhibition zone produced by the CFS using the agar diffusion assay, as described in Section 2.2. The growth curve was expressed in a graph by plotting the OD (605 nm) against the diameter of the inhibition zone registered over time. In addition, the pH was monitored during growth and plotted against the antimicrobial activity.

### 2.5. Partial Characterization of Antimicrobial Substances and In Vitro Stability

#### 2.5.1. Titrable Acidity and pH

The acidity was determined by titrating 25 mL of each CFS obtained as described in Section 2.2 with 0.1 N NaOH using phenolphthalein as an indicator [24]. Results were expressed as a percentage of lactic acid per 100 mL of CFS. Each of the measurements was carried out in triplicate using different batches of raw materials. The pH was measured by electrode immersion with a pH meter (S210, Mettler Toledo, Columbus, OH, USA) at 48 h of fermentation. Each experiment was performed in triplicate starting from individual yeast cultures.

#### 2.5.2. Enzymatic Sensitivity

To evaluate the chemical nature of the antimicrobial substances, the CFS was subjected to different treatments and the inhibitory activity was evaluated, as described in Section 2.2. Briefly, to rule out the possible inhibitory activity of organic acids, the CFS was heated at 80 °C for 10 min, the pH was adjusted to 6.0 (NCFS—neutralized CFS), and the activity was determined. In other experiments, the NCFS was treated with catalase enzyme (1 mg/mL) to prevent the possible inhibition of hydrogen peroxidase. Moreover, both the CFS and NCFS were treated individually with proteinase K (PK), pepsin, and alpha-chymotrypsin (Sigma-Aldrich Company, St. Louis, MO, USA) at final concentrations of 1 mg/mL, and subsequently incubated for 2 h at 37 °C and then for 5 min at 100 °C to inactivate the enzyme. Additionally, the CFS and NCFS were treated with lysozyme (Lys) at final concentrations of 1 and 3 mg/mL. Using *E. coli* ATCC25922 as the indicator strain, each agar assay experiment described in Section 2.2 was carried out in triplicate. A sterile M9 medium was used as a negative control. As a positive control, CFS without any treatment was employed.

#### 2.5.3. pH, Heat, and Detergent Sensitivity

Aliquots of CFS were incubated for 10, 30, and 60 min at 60, 80, 100, and 120 °C, as well as 15 min at 121 °C (autoclavation). In other batches, aliquots of CFS were adjusted to pH 2.0, 3.0, 6.0, 8.0, and 10.0, incubated for 2 h, and used in the agar diffusion assay. In addition, the effects of Triton X-100 (BDH Chemicals Ltd., Poole, UK), SDS (sodium dodecyl sulfate, Sigma-Aldrich Corporation, Burlington, WA, USA), and Tween 20 (Sigma-Aldrich Corporation, Burlington, WA, USA) at concentrations of 10 mg/mL were evaluated. Moreover, the CFS was treated with EDTA (Merck, Rahway, NJ, USA) at the permitted concentration of 0.1 mg/mL [25]. All experiments were run in triplicate using *E. coli* ATCC25922 as an indicator strain. As a negative control, the detected optimum growth medium was used. CFS without any treatment was used as a positive control. The percentage of residual antibacterial activity was calculated, as described in Section 2.3.

### 2.6. Inhibitory Spectrum

The inhibitory spectrum of the CFS was evaluated against several food pathogens, lactic bacteria, and some yeasts (laboratory CCMBIOGEM Collection) using the agar well diffusion method, as described in Section 2.2. The indicator strains used in this study are described in Table S1. In the other independent batches, the CFS was treated with Lys (3 mg/mL) and EDTA (0.1 mg/mL) and the activity was assessed against five target food pathogens (Table S1). Furthermore, in another experiment, the CFS was heated at 120 °C for 60 min and then used in the antimicrobial assay. Each experiment was performed in triplicate starting with the individual yeast cultures. The diameter of the inhibition zone was registered and the RIA was calculated, as indicated in Section 2.3.

### 2.7. Solvent Concentration and Partial Purification

The CFS obtained as described in Section 2.2 was treated with several solvents: (a) ammonium sulfate at 80% saturation (PPs); (b) ethyl acetate (*v*/*v*) (PPae); (c) methanol (*v*/*v*) (PPm). This was followed by 48 h incubation with refrigeration without stirring, and then centrifugation at 8000× *g* for 30 min at 4 °C. The PPs, PPae, and PPm were recovered in 25 mM ammonium acetate (pH 6.5), desalted using a midi dialysis kit (cat # PURD10005-1KT, Sigma-Aldrich Co. LLC, Saint Louis, MO, USA), pre-equilibrated with phosphate buffer (pH 7.0), dried for 48 h under the flow chamber, recuperated in sterile water, and stored at −20 °C. The inhibitory activity of each fraction was evaluated against *E. coli.* In addition, the impact of PPm alone, PPm + EDTA (0.1 mg/mL), and PPm + Lys (3 mg/mL) on *Salmonella, Listeria*, *E. coli*, *Staphylococcus*, and *K. cowanii* was evaluated, as described in Section 2.2 and 2.3. Moreover, the molecular weight was estimated by Tricine-SDS-PAGE electrophoresis. The precast acrylamide gels (15%) and a mini-vertical electrophoresis system (Expedeon Ltd., Abcam, Cambridge, MA, USA) were employed. The gel was stained with Bluestain ready-to-use (Sigma-Aldrich Co. LLC, Saint Louis, MO, USA) according to the manufacturer’s instructions.

### 2.8. Statistical Analysis

The results were reported as mean ± standard deviation. Kruskal–Willis one-way analysis of variance (non-parametric) and Tukey’s post hoc test were employed to identify significant differences between the means, and *p* < 0.05 was selected as the statistical significance level (SPSS version 10.0.6, USA, and Excel).

## 3. Results and Discussion

### 3.1. Medium Composition and Antimicrobial Activity

**Figure 1:**
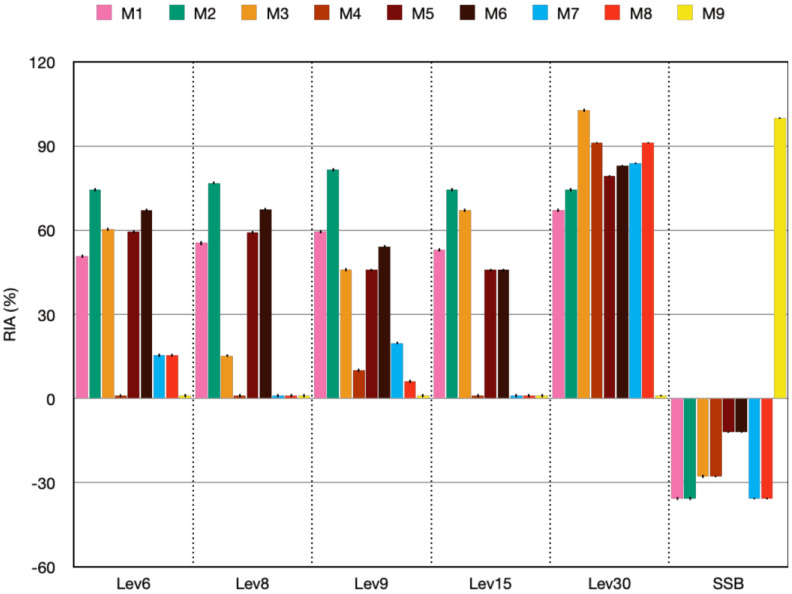
Residual activity (RIA%) of the target yeasts grown in different media. M1: M9 + 5% sucrose; M2: M9 + 10% sucrose; M3: M9 + 20% sucrose; M4: M9 + 40% sucrose; M5: M9 + 5% dextrose; M6: M9 + 10% dextrose; M7: M9 + 20% dextrose; M8: M9 + 40% dextrose; M9: YPD broth. RIA (%) = 1 − (Ac − As/Ac) × 100, where Ac is the inhibition zone of the control sample (M9 medium, no sugar added); As is the inhibition zone of the test sample (sugar-supplemented medium).

The supplementation of YPD with sucrose and dextrose led to an increase in the yield, which varied with the dose, type of sugar added, and strain, as no inhibitory activity was recorded when native yeasts were grown in the M9 medium (Table S2). Figure 1 displays the RIA (%) activity of yeasts in different media. Lev30 had a favorable inhibitory effect in all tested sugar-supplemented media, whereas Lev6, Lev8, Lev9, and Lev15 displayed high inhibitory activity in M1, M2, and M3 corresponding to 5%, 10%, and 20% sucrose. The inhibitory activity was absent in M4 (40% sucrose), except for Lev30. Similar results were obtained with the dextrose-supplemented medium. For the reference SSB, M9 was sufficient to achieve the maximum inhibitory effect, as the addition of sugars has a negative impact on the antimicrobial activity (Figure 1, Table S2). It is likely that the increased substrate concentration prevented fermentation because of osmotic stress (9). Recent studies on the growth response of four yeasts isolated from honey and floral nectar in a medium supplemented with glucose (2%, 10%, 20%, 40%, and 60%) revealed that glucose concentrations beyond 20% had a detrimental effect on cell growth [26]. In addition, SSB, a probiotic strain, showed inhibitory action against several gastrointestinal pathogens, including *Shigella flexneri, E. coli* (enteropathogenic and enterohaemorrhagic strains), *S. typhimurium*, and *C. albicans* [27,28]. In another study investigating the impact of various carbon source-supplemented media (such as glucose, sucrose, fructose, etc.) on the antimicrobial activity of yeasts isolated from different fruits, the CFS obtained from glucose-supplemented medium induced the highest antimicrobial activity against other pathogenic yeasts but not bacteria [29].

Our research revealed that native yeasts had a strong antibacterial effect against *E. coli* in all sugar-supplemented media examined. However, the M2 medium (10% sucrose) was chosen for the subsequent experiments since it provided a comparable level of inhibitory activity for all target yeasts. We concluded that the dosage and type of the carbon source added to the basal medium play a crucial role in yeast growth by triggering fermentation and promoting the production of complex compounds with inhibitory capacity. However, more research is needed to understand the mechanism of action under high sugar content.

### 3.2. Growth Kinetics and Antimicrobial Activity

**Figure 2:**
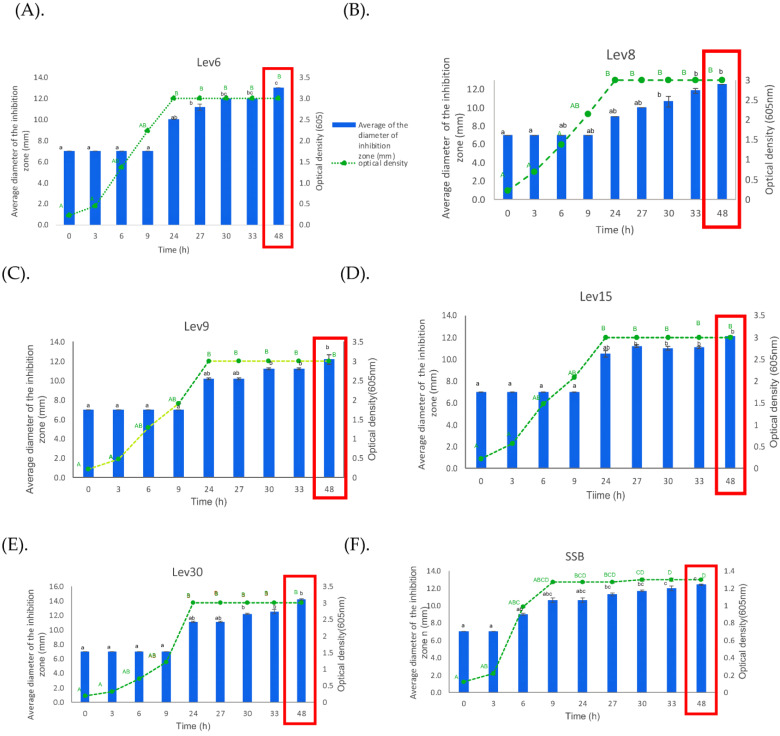
Cell growth expressed as the OD (605 nm) and inhibitory activity (diameter of the inhibition zone) monitored for 48 h for (**A**) Lev6; (**B**) Lev8; (**C**). Lev9; (**D**) Lev15; (**E**) Lev30; (**F**) SSB. Data are means ± standard error. Values with different letters are significantly different at *p* < 0.05. Lowercase letters indicate differences within the inhibition zone–incubation time, and uppercase letters indicate differences within the optical density–incubation time. The bars signify the inhibition zone. The 6.0–6.5 mm values indicate no activity. The red box indicates the maximum inhibitory zone.

The production and growth rate of antimicrobial substances were assessed in the M2 medium during 48 h of incubation at 30 °C and 200 rpm. The growth values were plotted against the diameter of the inhibition zone over time (Figure 2A–E). The results indicated that native yeasts adapted very effectively in the M2 medium and that antimicrobial substances were produced following 24 h of growth. Fermentative growth is typically characterized by a high specific growth rate and a low duplication time, whereas respiratory growth is typically characterized by a low specific growth rate and a high multiplying period [30]. In addition, the pH gradually decreased over time, reaching its lowest value at 24 h, and then remained stable for another 48 h (Figure S1A–E). This can be attributed to the acceleration of yeast growth and the production of inhibitory substances. For all native yeasts, the maximal inhibitory activity was achieved at 48 h when the pH dropped from 6.23 to 4.18 (Figure S1A–E). Instead, the reference SSB showed early production of antimicrobial substances starting at 9 h of cell growth in the M9 medium, which coincided with a reduction in pH from 6.60 to 4.59. In addition, the optical density was 1.3 and the inhibitory zone was 12.17 ± 0.28 mm at 48 h (Figure 2F). In previous studies, the maximum antibacterial activity against *B. subtilis*, *S. aureus*, *E. coli*, and *Klebsiella aerogenes* was obtained when yeasts were grown at 25–30 °C for 48 h [7]. Based on these results, we speculate that during the fermentation of sugars, native yeasts produce antimicrobial-like substances, such as acids, toxins, peptides, proteases, and other substances, which may be responsible for the inhibitory activity against *E. coli.* Complementary analysis indicated that Lev6, Lev8, Lev30, and the reference SSB, but not Lev9 and Lev15, displayed proteolytic activity in skim milk (1%) agar-containing medium (clear zone around the colonies), suggesting that they may produce extracellular proteases that contribute to the overall antimicrobial capacity. This feature might be advantageous if these yeasts are intended for use in fermentation processes involving high-sugar-content raw materials such as fruits or cereals. The compounds released by these yeasts during fermentation may help to protect the final product against pathogenic bacteria.

### 3.3. Evaluation of Antimicrobial Substances and Partial Characterization

#### 3.3.1. Acidity and pH Evaluation

**Figure 3:**
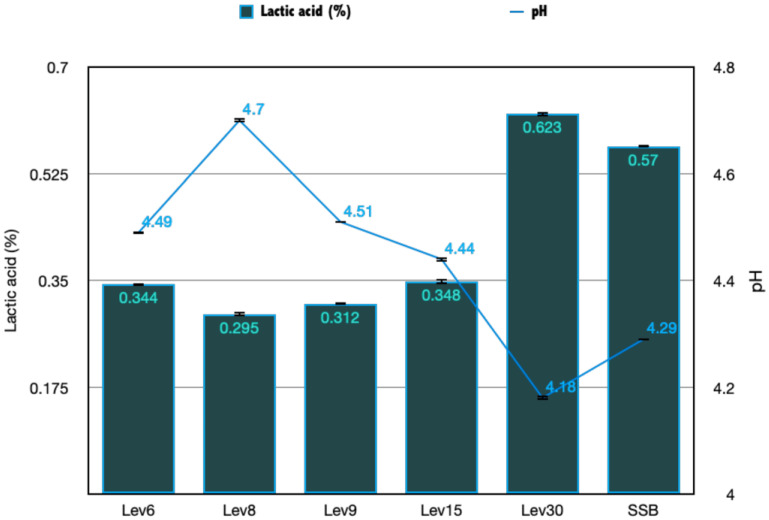
pH and acidity expressed as % of lactic acid assessment of CFS after 48 h of fermentation. Data are means ± standard error. CFS: cell-free supernatant.

The ability of yeasts to reduce the growth of harmful bacteria is related to the production of inhibitory metabolites, such as organic acids, diacetyl, and peptides, according to previous research [29]. In this investigation, Lev30 and SSB showed the highest acidity after 48 h of fermentation, which correlated with the pH drop at 4.18 for Lev30 and 4.29 for SSB, respectively (Figure 3). In addition, these strains showed the highest antagonistic activity against *E. coli*. These findings are consistent with previous studies indicating that yeasts produce metabolites capable of modifying the rigidity and structure of the target cell wall, causing its breakdown. Therefore, acids play a key role in the antagonistic ability of yeast species against target bacteria or fungi [31]. The cell structure and secondary metabolites of antagonistic yeasts may undergo changes that increase disease resistance. *C. saitoana*, an antagonistic yeast, has been shown by El-Ghaouth et al. [32] to cause host cell deformation, produce mastoid structures, and subsequently prevent *Botritys cinerea* infection. Further analyses are required to determine the compositions of these extracts.

#### 3.3.2. Enzyme Sensitivity

The average values of the inhibitory zones following treatment with enzymes are shown in Table S3. The treatment of the CFS at physiological pH with PK resulted in a statistically significant (*p* < 0.05) reduction in activity, suggesting that the compounds released in the supernatant may be attributed to the production of proteinaceous substances, acids, or volatile compounds (Table S3). PK cleaves peptide chains primarily at the peptide bond close to the carboxyl group of aliphatic and aromatic amino acids with blocked alpha amino groups [29]. At a neutralized pH (6.0) (NCFS) and when treated with catalase, the antimicrobial activity was abolished, suggesting that the activity may be dependent on hydrogen peroxide. However, the activity was maintained when the CFS was treated with the indicated enzymes (Table S3). No activity was observed when the NCFS was treated with enzymes. At concentrations of 1 and 3 mg/mL, Lys alone did not exert an antimicrobial effect against *E. coli*, whereas the pretreatment of the CFS obtained from Lev30 and SSB with Lys at 3 mg/mL showed an increase in inhibitory action against *E. coli.* No changes in activity were seen with the other yeasts. Previous studies have indicated that Lys is ineffective against Gram-negative bacteria because of their thin layer of peptidoglycan without teichoic acid, which is covered by an outer membrane that acts as a barrier and limits the access of Lys to peptidoglycan chains [33,34]. Nonetheless, this barrier can be disrupted by membrane-permeabilizing agents such as organic acids and EDTA. Additionally, conjugation of Lys with carbohydrates, denaturation of lysozyme, and modification of lysozyme by linking it to other compounds such as polysaccharides, hydrophobic peptides, and fatty acids have been shown to overcome this limitation [35]. Based on these results, we suggest that the inhibitory activity may depend, in part, on the acidity, hydrogen peroxide, and other inhibitory substances released from the CFS (proteases, proteins, peptides). It is also important to note that the overall activity of the target yeasts is species-dependent.

#### 3.3.3. Effect of pH, Heat, and Detergent Exposure on Antimicrobial Activity

**Figure 4:**
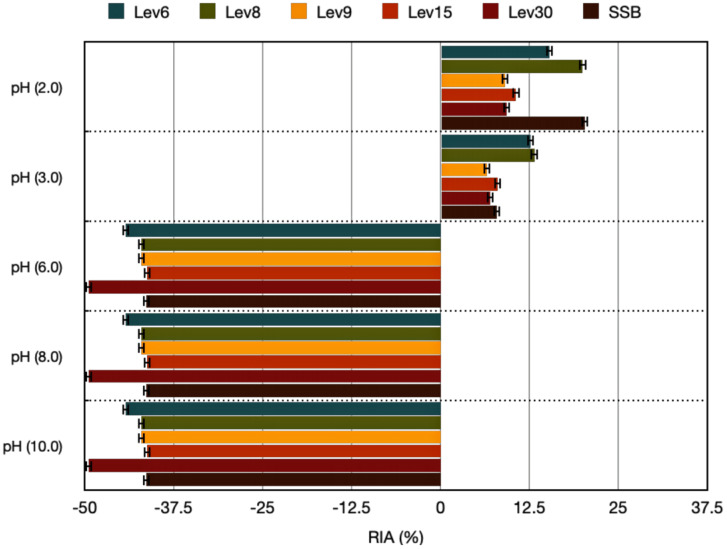
Effect of pH on residual activity (RIA %) against *E. coli* ATCC25922. RIA (%) values were calculated relative to CE from yeasts without any treatment. The results are representative of three independent experiments. RIA (%) = 1 − (Ac − As/Ac) × 100, where Ac is the inhibition zone of the control sample (CFS). CFS: cell-free supernatant.

The highly acidic conditions (pH 2.0) led to increased inhibitory activity against *E. coli* for each of the yeasts tested (Figure 4), indicating that acidity can increase inhibitory activity through increased protein or peptide solubility. Additionally, acids may be able to cross the membranes of target cells, acidifying the cytoplasm and improving its permeability [36]. The activity was maintained at pH 3.0 for each of the target yeasts. In addition, the neutral pH did not have a positive impact on the inhibitory activity. These results align with previous studies indicating that the CFS at pH 4.0 showed optimal antimicrobial activity against Gram-positive bacteria, whereas at neutral pH, the activity was abolished [29,37]. However, in our study, the inhibitory action against *E. coli* was positively regulated by acidity, whereas alkaline media were detrimental to the inhibitory activity. This property limits the usage of alkaline food matrices. Additionally, following exposure of the CFS to different temperatures and incubation times, the inhibitory activity was maintained, suggesting that the active compounds released in the CFS are thermostable (Figure S2). An increase was observed at 120 °C after 60 min, suggesting that the compounds released in the CFS may be better solubilized and concentrated by heat. We propose that a thermally induced chemical interaction (Maillard’s reaction), which involves active components such as amino and carbonyl groups, may contribute to the observed increase in inhibitory activity following heat exposure [38]. The effectiveness of Maillard reaction products with inhibitory action against pathogens has been reported in previous studies with LAB [39]. These properties may be related to the high molecular weight of the released proteins, which can bind chemical elements such as iron, copper, or zinc, thereby increasing the antimicrobial effect [40]. A previous study reported that heating the CFS of yeasts isolated from dairy products resulted in increased inhibitory activity against *Listeria*. This may have been due to the hydrophobic heat-resistant peptides that induced pore formation in the cell envelope and resulted in the loss of cytoplasm [40]. Nonetheless, our results contradict those of previous studies where the optimum antimicrobial activity for some yeasts was detected at 30 °C, suggesting that the increased temperature of 45 °C has a detrimental effect on antimicrobial action [29]. In our study, the increased temperature and incubation time positively influenced the antimicrobial action. However, the heat activation of antimicrobials produced by yeasts could be an advantage for those strains intended to be used in heat-derived food products. Furthermore, the impact of different chelating agents on the inhibitory activity of the CFS derived from native yeasts was assessed. However, EDTA treatment showed a positive impact on the antimicrobial activity of the native yeasts and reference SSB (Figure S3). Triton-X100, SDS, and Tween20 had a negative impact on the antimicrobial activity of all target yeasts; however, Triton-X100 enhanced the antimicrobial activity of SSB. No inhibition was observed when treated with each chemical substance alone. The effect of chelating agents on crude extracts from different LAB strains has been intensely studied against Gram-negative bacteria [41]. This activity has been associated with an increase in the permeability of the outer membrane that goes beyond cation extraction (Ca^2+^, Mg^2+^), allowing antimicrobial peptides to enter the cytoplasmic membrane. The favorable effect on inhibitory activity was shown to be strain-dependent. However, our study is the first to examine the impact of such compounds on antimicrobial substances derived from yeasts. The antimicrobial efficacy against *E. coli* was positively influenced by high heat (120 °C, 60 min), acidic conditions (pH = 2.0), and, to a lesser extent, EDTA, according to our findings.

### 3.4. High Heat, EDTA, and Lys-Enhanced Antimicrobial Action against Some Food Pathogens

**Figure 5:**
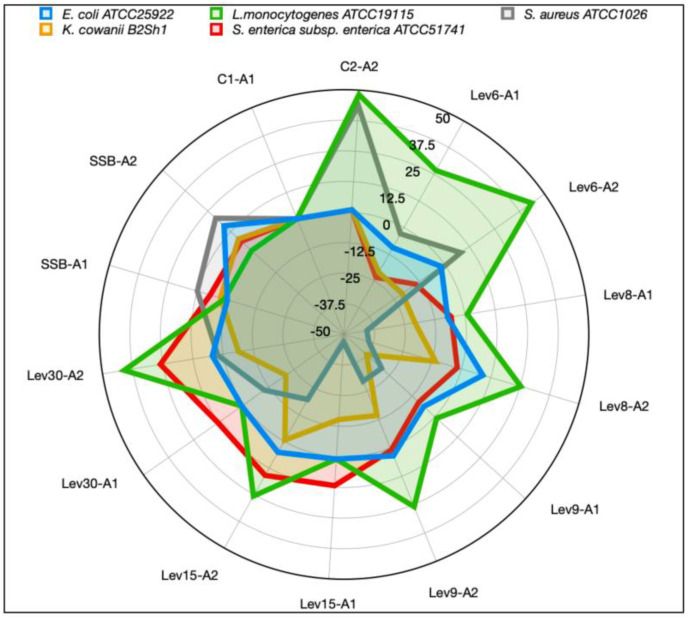
Radar plot representation of RIA (%) following exposure of CE to Lys against three Gram-negative (*E*. *coli*, *K*. *cowanii,* and *S. enterica*) and two Gram-positive (*S*. *aureus*, *L. monocytogenes*) bacteria. The results are representative of three independent experiments. RIA (%) = 1 − (Ac − As/Ac) × 100, where Ac is the inhibition zone of control sample (CFS); As is the inhibition zone of the test sample (CFS + Lys). C1: control Lys (1 mg/mL); C2: control Lys (3 mg/mL); A1: 1 mg/mL; A2: 3 mg/mL; CFS: cell-free supernatant.

Differential activity was seen against *Salmonella, Listeria*, *E. coli*, *Staphylococcus*, and *K. cowanii* B2Sh1 following the heat treatment (120 °C, 60 min) of both the CFS and PPm forms (Table 1). The outcomes demonstrated that the action was pathogen- and strain-dependent. Among the yeasts tested, Lev30 in the PPm form showed a statistically significant increase (*p* < 0.05) in antimicrobial activity against *Salmonella* and *Staphylococcus.* PPm was obtained through precipitation of the CFS extracted following the growth of yeasts in 20% sugar. At high temperatures, the protein structure may be affected, which could result in variations in protein cross-linking and the occurrence of the Maillard reaction [42]. In addition, the Maillard reaction is related to carbohydrates and proteins so the level of antimicrobial activity depends on the sugar fermentation and protein modification following heat treatment [43]. Previous studies have indicated that the exposure of the wine-spoilage yeast *B. bruxellensis* to the supernatant of *C. intermedia* LAMAP1790 at 4 °C, but not at 100 °C, resulted in the inhibition of these spoilage yeasts, with this activity being attributed to a heat-labile peptide [6]. In addition, some yeasts produce mycocins that suppress unwanted bacteria, yeasts, and molds [44,45]. Among them, *C. catenulata*, *C. parapsilosis*, *C. tropicalis, Debaryomyces hansenii*, *Geotrichum candidum*, *Pichia fermentans*, and *P. anomala*, showed significant inhibitory effects against *Listeria ivanovii* HPB28 in an agar-membrane screening test [46]. Moreover, Lev6 and SSB, as well as Lev9, Lev8, and Lev30, but not Lev15, displayed positive inhibitory effects against *Staphylococcus* following the pretreatment of CFS with EDTA (0.1 mg/mL) (Figure S4). Lev8, Lev15, and Lev30 inhibited *K. cowanii* B2Sh1, whereas CFS with EDTA boosted the inhibitory effect of all target yeasts against *E. coli*. Although Lev30 and SSB exhibited maximum activity against *Salmonella* following pretreatment with EDTA, there was no effect against *Listeria.* This implies that the inhibitory activity depends on the strain strength and target pathogen. It is not novel to use EDTA as a potentiating and sensitizing agent, but when used synergistically with other antimicrobials, the ability to manage biofilms may be significantly improved [47]. These results support earlier research evaluating the effect of some yeasts isolated from cattle products on harmful bacteria such as *P. aeruginosa*, *E. coli*, and *S. aureus* [48]. In addition, the effect of CFS pretreatment with Lys (3 mg/mL) was evaluated against *Listeria*, *Staphylococcus*, *E. coli*, *Salmonella*, and *K. cowanii.* Lev8, Lev15, and Lev30 had a favorable effect on *Salmonella*, whereas Lev30 and SSB had strong activity against *K. covanii* (Figure 5). At higher concentrations (3 mg/mL), Lys alone showed activity against *Listeria* and *Staphylococcus* only. An inhibitory effect against *Listeria* was achieved when a higher dose of Lys (3 mg/mL) was mixed with the CFS of Lev6, Lev30, and SSB, but no activity was seen at the lower dose (1 mg/mL) (Figure 5). Since lysozyme is a cationic protein, it may create gaps in the bacterial membrane through electrostatic interactions with phospholipids, causing cell lysis due to membrane leaking without peptidoglycan degradation [34,35]. The electrostatic reaction that occurs inside the cell membrane gives the antimicrobial peptides produced by yeast cells their high level of antagonistic activity [7]. The secretion of various substances during fermentation, such as volatile thermolabile toxic extracts, proteases, mycocins, or peptides, may be the cause of the inhibitory action against the utilized bacterial species [7,23]. Overall, the heat treatment showed a positive impact on the inhibitory effect against both Gram-positive and -negative bacteria, indicating the proteinaceous nature of the compounds released in the supernatant. Further investigations are required to identify these metabolites, as well as their mode of action against pathogenic and biofilm formation strains.

### 3.5. Estimation of Molecular Weight and Antimicrobial Activity of Concentrated and Partially Purified Fractions

**Figure 6:**
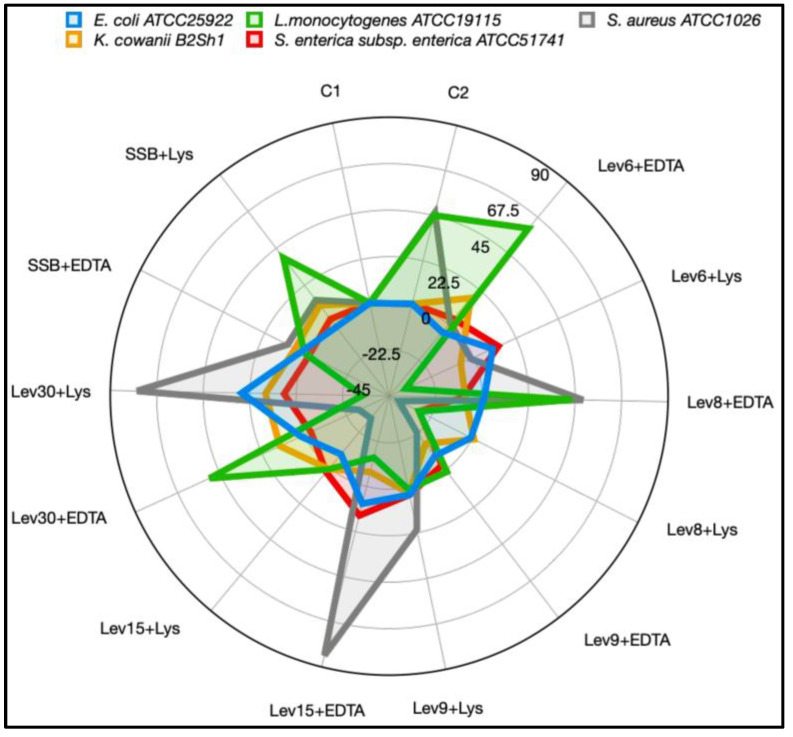
Radar plot representation of RIA (%) following exposure of PPm to EDTA and Lys against three Gram-negative (*E*. *coli*, *K*. *cowanii*, and *S. enterica*) and two Gram-positive (*S*. *aureus*, *L. monocytogenes*) bacteria. The results are representative of three independent experiments. RIA (%) = 1 − (Ac − As/Ac) × 100, where Ac is the inhibition zone of control sample (PPm); As is the inhibition zone of the test sample (PPm + Lys; PPm + EDTA). C1: control EDTA (0.1 mg/mL); C2: control Lys (3 mg/mL); PPm: extracellular fractions obtained by precipitating with methanol.

**Figure 7:**
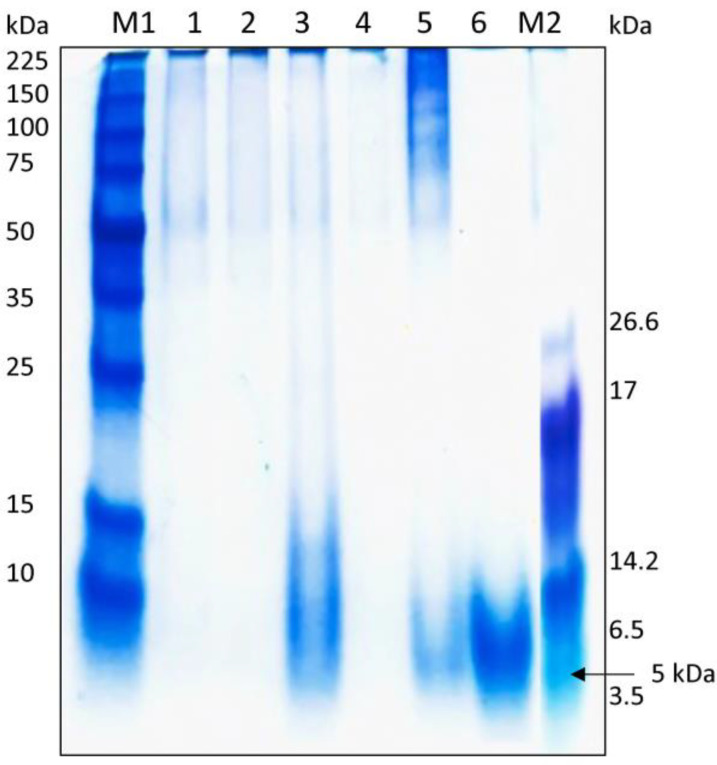
Tricine-SDS PAGE profile of PPm obtained from yeasts. Lane M1: low-range protein ladder (Promega, # V8491); Lane 1: PPmLev6; Lane 2: PPMLev8; Lane 3: PPmLev9; Lane 4: PPmLev15; Lane 5: PPmLev30; Lane 6: PPmSSB; Lane M2: color marker ultra-low-range marker (Sigma, # C6210-1VL).

Initial tests for the antimicrobial activity against *E. coli* were conducted with the partially purified substances produced by the precipitation of the CFS with ammonium sulfate (PPs), ethyl acetate (PPae), and methanol (PPm). A high inhibitory effect was seen with PPm, whereas reduced activity was identified with PPs and PPae indicating that methanol precipitated active fractions of a proteinic origin. The presence of an additional membrane permeabilizer, as well as the complex chemical combination precipitated with a particular solvent, boosted the overall efficacy against some pathogens, as evidenced by the treatment with EDTA (Figure 6). Compared to its CFS equivalent, PPm alone had a moderate impact on *Salmonella*, *Staphylococcus*, and *Kosakonia* (Figure 6). PPm + Lys had no activity against *Listeria* in contrast to the results with the CFS (Figure 5), whereas the PPm of Lev6, Lev8, and Lev30 in combination with EDTA had a favorable inhibitory effect. An increase in activity was observed for the PPm of Lev30 + Lys against *Staphylococcus.* Interestingly, the PPm combined with Lys increased the activity against *Salmonella* for all target yeasts, suggesting a synergistic effect, as Lys alone did not inhibit *Salmonella*. In addition, the PPm of Lev9 contained molecules of about 10 kDa (Figure 7, Lane 3), whereas the PPm of Lev30 and SSB contained peptides with molecular masses under 5 kDa (Figure 7, Lane 5, Lane 6). The strong band of SSB may be related to the amount of lysine and arginine amino acid content of the molecule since the pH of the running buffer was 8.3. Based on the number of positive charges present on the protein, Coomassie binds to basic amino acids in a nearly proportionate manner [49]. A heat-labile peptide under 5 kDa, produced by *C. intermedia* LAMAP1790 and active against the wine-spoilage yeast *B. bruxellensis,* was detected using a silver stain [6]. On the upper side of the gel, high-molecular masses (35–50 kDa) and very faint bands were observed for Lev6, Lev8, and Lev15. Early research indicated that *C. pyralidae* produces antibacterial toxins of high-molecular mass capable of biocontrolling several *B. bruxellensis* strains [50]. However, those molecules may lack arginine and lysine, thus other stain alternatives such as zinc or silver should be used to detect proteins with different binding specificity. Nonetheless, these fractions need to be examined more thoroughly to identify their amino acid compositions. These results are consistent with past studies, showing that a mixture of several compounds is responsible for the overall antibacterial strength [9,51]. This could be advantageous because manufacturing at a larger scale will increase overall costs when applied to food products.

## 4. Conclusions

To the best of our knowledge, this is the first study demonstrating the antibacterial activity against both Gram-positive and Gram-negative bacteria of compounds derived from yeasts isolated from tropical berries from Ecuador. Highly thermostable antimicrobials were detected. The results indicated that the level of inhibitory action was dependent on the yeast strains, their metabolites produced during sugar fermentation, and possible protein alterations caused by heat (occurrence of Maillard reaction). The antilisterial effect was obtained when PPm was combined with a membrane-permeable agent (EDTA). In addition, the inhibitory effect on Gram-negative bacteria increased when PPm was pretreated with lysozyme. *S. cerevisiae* Lev30 was found to be one of the most efficient yeast strains. Further analyses are required to ascertain the mode of action and chemical composition of the inhibitory metabolites.

## Figures and Tables

**Table 1:** Effect of high heat on inhibitory activity against Gram-positive and Gram-negative bacteria.

Strain	Treatment	*E. coli* ATCC25922	*L. monocytogenes* ATCC19115	*S. aureus* ATCC1026	*K. cowanii* B2Sh1	*S. enterica* subsp. *enterica* ATCC51741
Lev6	CFS (RT)	12.83 ± 0.20 ^e^	7.02 ± 0.10 ^c^	9.02 ± 0.02 ^g^	10.67 ± 0.45 ^f^	11.01 ± 0.02 ^f^
	CFS (120 °C, 60 min)	13.70 ± 0.29 ^cd^	7.02 ± 0.10 ^c^	12.50 ± 0.29 ^de^	11.67 ± 0.29 ^e^	12.10 ± 0.29 ^e^
	PPm (120 °C, 60 min)	13.80 ± 0.29 ^cd^	7.02 ± 0.10 ^c^	13.33 ± 0.29 ^d^	13.33 ± 0.29 ^cd^	13.80 ± 0.29 ^d^
Lev8	CFS (RT)	12.33 ± 0.29 ^ef^	7.02 ± 0.10 ^c^	12.01 ± 0.29 ^e^	11.51 ± 0.29 ^ef^	12.67 ± 0.45 ^de^
	CFS (120 °C, 60 min)	13.51 ± 0.29 ^cd^	7.02 ± 0.10 ^c^	11.01 ± 0.29 ^ef^	12.51 ± 0.29 ^d^	13.51 ± 0.29 ^d^
	PPm (120 °C, 60 min)	14.81 ± 0.29 ^c^	7.02 ± 0.10 ^c^	14.81 ± 0.29 ^bc^	10.81 ± 0.29 ^f^	16.51 ± 0.29 ^b^
Lev9	CFS (RT)	12.17 ± 0.29 ^f^	7.02 ± 0.10 ^c^	11.83 ± 0.23 ^ef^	12.67 ± 0.25 ^d^	11.67 ± 0.15 ^ef^
	CFS (120 °C, 60 min)	13.50 ± 0.20 ^cd^	7.02 ± 0.10 ^c^	11.01 ± 0.30 ^ef^	12.33 ± 0.29 ^de^	13.51 ± 0.29 ^d^
	PPm (120 °C, 60 min)	13.50 ± 0.20 ^cd^	7.02 ± 0.10 ^c^	14.81 ± 0.30 ^bc^	15.33 ± 0.29 ^b^	16.51 ± 0.29 ^b^
Lev15	CFS (RT)	12.16 ± 0.20 ^f^	7.02 ± 0.10 ^c^	12.83 ± 0.36 ^d^	11.02 ± 0.10 ^d^	11.01 ± 0.01 ^f^
	CFS (120 °C, 60 min)	13.16 ± 0.15 ^d^	7.02 ± 0.10 ^c^	12.16 ± 0.29 ^de^	13.33 ± 0.51 ^cd^	12.66 ± 0.45 ^de^
	PPm (120 °C, 60 min)	13.16 ± 0.15 ^d^	7.02 ± 0.10 ^c^	14.31 ± 0.29 ^c^	13.22 ± 0.51 ^cd^	15.51 ± 0.28 ^c^
Lev30	CFS (RT)	14.17 ± 0.29 ^c^	7.02 ± 0.10 ^c^	12.33 ± 0.58 ^de^	13.67 ± 0.29 ^c^	11.33 ± 0.58 ^ef^
	CFS (120 °C, 60 min)	15.67 ± 0.29 ^b^	7.02 ± 0.10 ^c^	12.83 ± 0.28 ^de^	12.01 ± 0.30 ^de^	17.66 ± 0.29 ^a^
	PPm (120 °C, 60 min)	15.67 ± 0.29 ^b^	8.02 ± 0.10 ^c^	16.21 ± 0.28 ^b^	15.21 ± 0.28 ^b^	16.66 ± 0.29 ^b^
SSB	CFS (RT)	12.67 ± 0.17 ^ef^	7.02 ± 0.10 ^c^	10.61 ± 0.30 ^f^	11.64 ± 0.25 ^e^	11.33 ± 0.10 ^ef^
	CFS (120 °C, 60 min)	13.01 ± 0.17 ^e^	11.02 ± 0.10 ^a^	12.51 ± 0.28 ^e^	11.21 ± 0.29 ^ef^	13.51 ± 0.28 ^e^
	PPm (120 °C, 60 min)	18.01 ± 0.29 ^a^	9.71 ± 0.29 ^b^	19.01 ± 0.28 ^a^	16.51 ± 0.17 ^a^	17.21 ± 0.17 ^ab^

Data are means ± standard error. Values in the same column with lowercase letters are significantly different (*p* < 0.05). RT: room temperature; CFS: cell-free supernatant; PPm: extracellular fractions obtained by precipitating with methanol.

## Data Availability

The data used to support the findings of this study can be made available by the corresponding author upon request.

## Supplementary Materials

The following supporting information can be downloaded at: https://www.mdpi.com/article/10.3390/foods12132435/s1, Figure S1: pH variation during growth and antimicrobial activity; Figure S2: Radar plot representation of RIA (%) following exposure of CFS to different temperatures and times against *E. coli*; Figure S3: Radar plot representation of RIA (%) following treatment of CFS with different compounds against *E. coli*; Figure S4: Effect of CFS combined with EDTA (0.1 mg/mL) against three Gram-negative (*E. coli*, *K. cowanii*, and *Salmonella enterica*) and two Gram-positive (*S. aureus, L. monocytogenes*) bacteria; Table S1: List of indicator strains used in this study; Table S2: Effect of culture media on antimicrobial activity; Table S3: Effect of enzymes on antimicrobial activity against *E. coli.*
