# Supplementary material for: Inhibitory-like Substances Produced by Yeasts Isolated from Andean Blueberries: Prospective Food Antimicrobials
Source: Foods. 2023 Jun 21;12(13):2435. doi: 10.3390/foods12132435 (PMC10340612; doi:10.3390/foods12132435)

Figure S1. pH variation during growth and antimicrobial activity. (A). Lev6; (B). Lev8; (C). Lev9; (D). Lev15; (E). Lev30; (F). SSB. Data are means ± standard error. Values with different letters are significantly different *P* < 0.05. Small letters show the difference within the inhibition zone-incubation time; Capital letter show the differences within the pH-incubation time. The bars signify the inhibition zone. The 6.0-6.5 mm values indicate no activity.

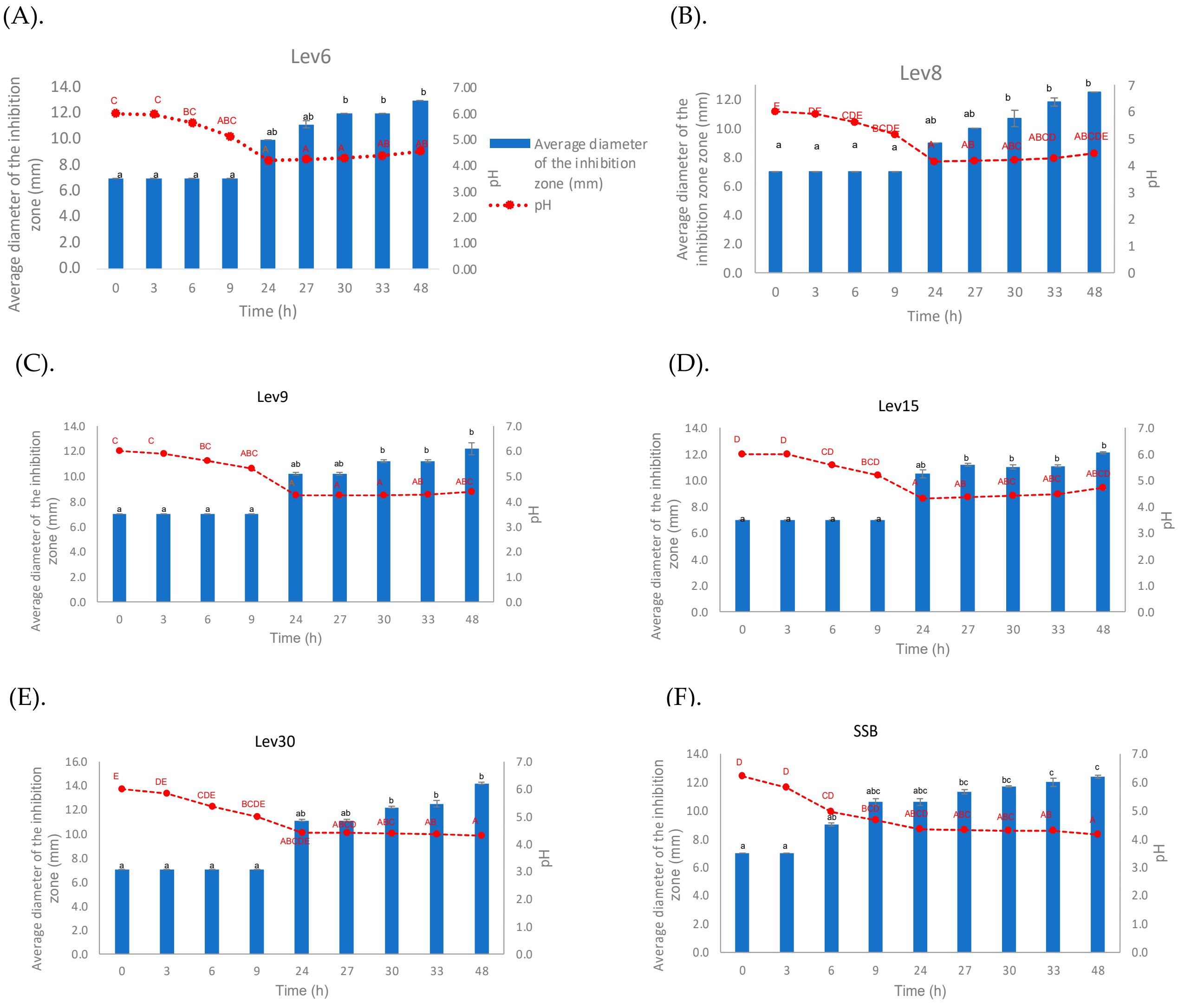

Supplement: Supplementary file 1 [file foods-12-02435-s001.zip › foods-2445305-supplementary-Figure S1.pdf]
