# Supplementary material for: Inhibitory-like Substances Produced by Yeasts Isolated from Andean Blueberries: Prospective Food Antimicrobials
Source: Foods. 2023 Jun 21;12(13):2435. doi: 10.3390/foods12132435 (PMC10340612; doi:10.3390/foods12132435)

Figure S2. RIA (%) radar plot representation upon exposure of CFS at different temperatures and times towards *E. coli*. The results are representative of three independent experiments. RIA (%) values were calculated relative to CFS from yeasts without any treatment. RIA (%) = 1− (Ac − As/Ac) × 100, where Ac is the inhibition zone of control sample (CFS); As is the inhibition zone of test sample (CFS + Lys); CFS: cell-free supernatant.

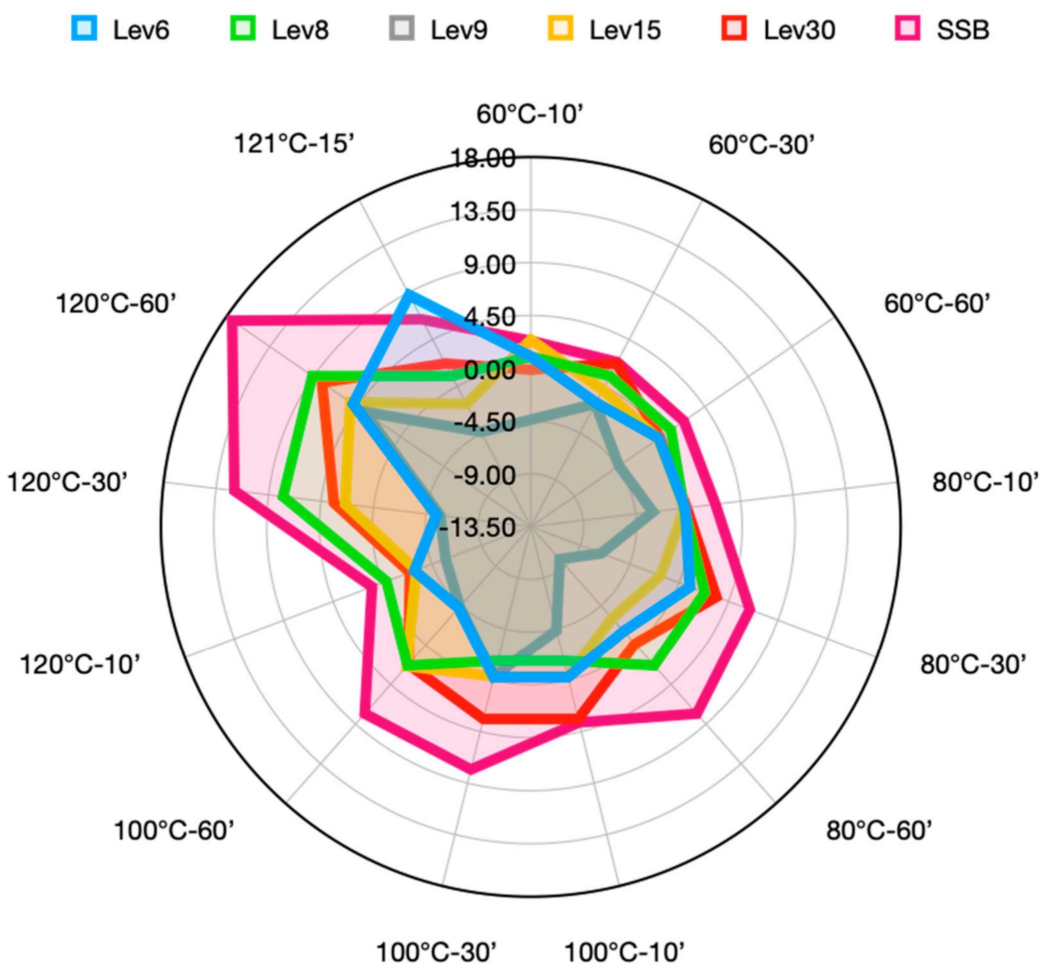

Supplement: Supplementary file 1 [file foods-12-02435-s001.zip › foods-2445305-supplementary-Figure S2.pdf]
