# Supplementary material for: Inhibitory-like Substances Produced by Yeasts Isolated from Andean Blueberries: Prospective Food Antimicrobials
Source: Foods. 2023 Jun 21;12(13):2435. doi: 10.3390/foods12132435 (PMC10340612; doi:10.3390/foods12132435)

Figure S3. RIA (%) radar plot representation upon the treatment of CFS with different compounds towards *E. coli*. The results are representative of three independent experiments. RIA (%) values were calculated relative to CFS from yeasts without any treatment. RIA (%) = 1− (Ac − As/Ac) × 100, where Ac is the inhibition zone of control sample (CFS); As is the inhibition zone of test sample (CFS + each compound). C1: SDS 10 (mg/mL); C2: Triton-X (10 mg/mL); C3: Tween (10 mg/mL); C4: EDTA (0.1 mg/mL).

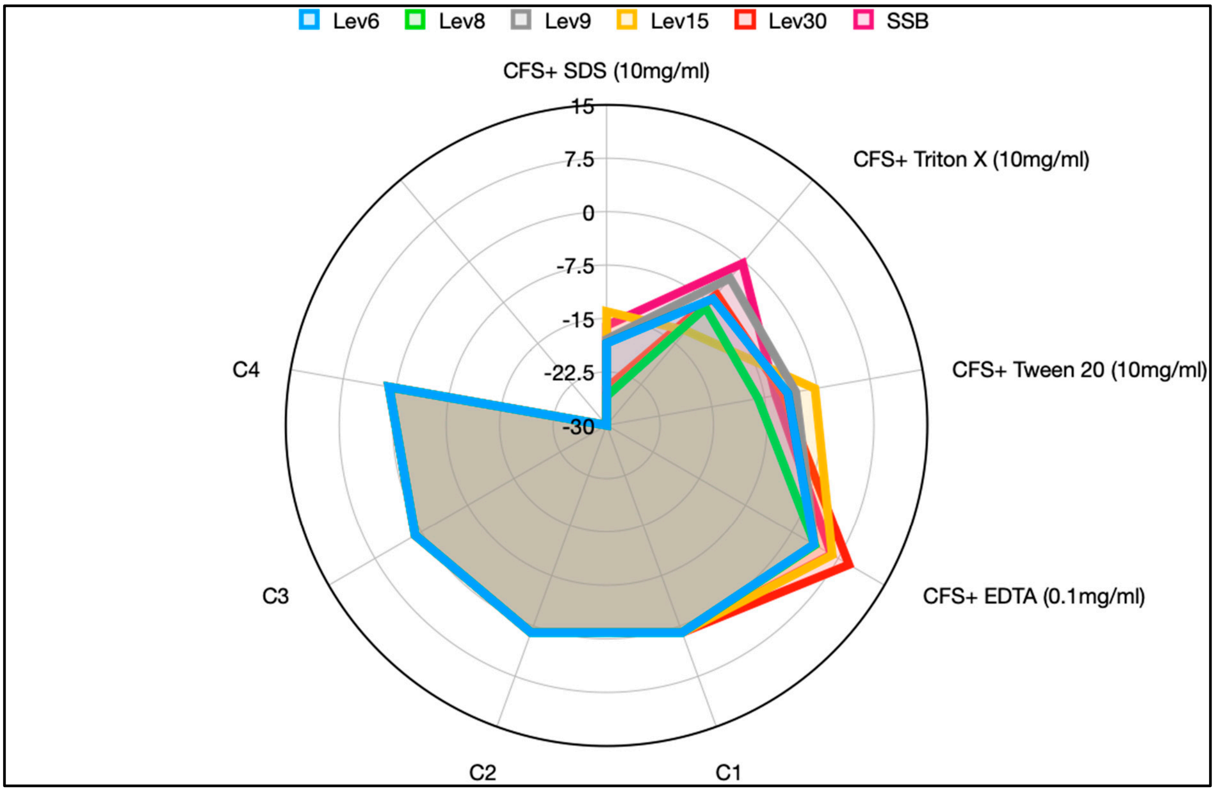

Supplement: Supplementary file 1 [file foods-12-02435-s001.zip › foods-2445305-supplementary-Figure S3.pdf]
