# Supplementary material for: Inhibitory-like Substances Produced by Yeasts Isolated from Andean Blueberries: Prospective Food Antimicrobials
Source: Foods. 2023 Jun 21;12(13):2435. doi: 10.3390/foods12132435 (PMC10340612; doi:10.3390/foods12132435)

Figure S4. Effect of CFS combined with EDTA (0.1mg/mL) on three Gram-negative (*E. coli*, *K. cowanii* and *Salmonella enterica*) and two Gram-positive (*S. aureus*, *L. monocytogenes*) bacteria. The results are representative of three independent experiments. RIA (%) values were calculated relative to CFS without treatment. RIA (%) = 1− (Ac − As/Ac) × 100, where Ac is the inhibition zone of control sample (CFS); As is the inhibition zone of test sample (CFS + EDTA); CFS: cell-free supernatant.

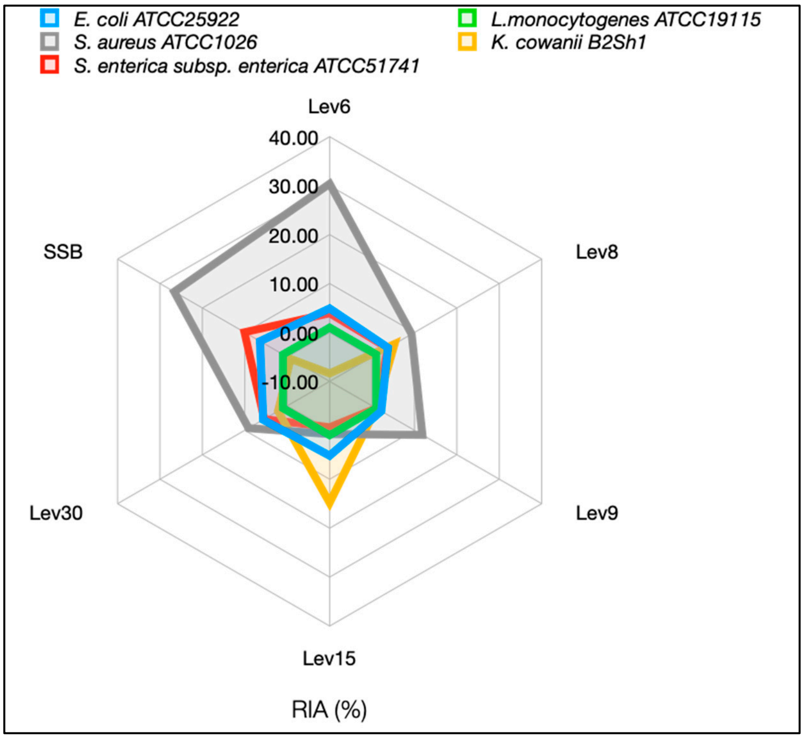

Supplement: Supplementary file 1 [file foods-12-02435-s001.zip › foods-2445305-supplementary-Figure S4.pdf]
