# Supplementary material for: Inhibitory-like Substances Produced by Yeasts Isolated from Andean Blueberries: Prospective Food Antimicrobials
Source: Foods. 2023 Jun 21;12(13):2435. doi: 10.3390/foods12132435 (PMC10340612; doi:10.3390/foods12132435)
Supplement: Supplementary file 1 [file foods-12-02435-s001.zip › foods-2445305-supplementary-Table S1.pdf]

Table S1. List of indicator strains used in this study.

**Indicator strains**
E. coli ATCC25922
Listeria monocytogenes ATCC19115
Staphylococcus aureus ATCC1026
Shigella dysenteriae UTNFa37-1
Kosakonia cowanii B2Sh1
Salmonella enterica subsp. enterica ATCC51741
Lactococcus lactis ATCC11474
Lactobacillus reuteri DSM17938
Saccharomyces spp. B1Lev2 (laboratory isolate)
Saccharomyces spp. B2Lev2 (laboratory isolate)
Saccharomyces spp. B2Lev1 (laboratory isolate)
Saccharomyces spp. B1Lev4 (laboratory isolate)
