# Supplementary material for: Inhibitory-like Substances Produced by Yeasts Isolated from Andean Blueberries: Prospective Food Antimicrobials
Source: Foods. 2023 Jun 21;12(13):2435. doi: 10.3390/foods12132435 (PMC10340612; doi:10.3390/foods12132435)
Supplement: Supplementary file 1 [file foods-12-02435-s001.zip › foods-2445305-supplementary-Table S2.pdf]

Table S2. Effect of culture media on antimicrobial activity.

Medium description	Code medium	Concentration of carbohydrate	Average diameter of the inhibition zone (mm)					
			**Lev6**	**Lev8**	**Lev9**	**Lev15**	**Lev30**	**SSB**
**YPD + sucrose**	M1	5%	10.51 ± 0.2^{b}	10.83 ± 0.3^{c}	11.12 ± 0.2^{ab}	10.66 ± 0.2^{b}	11.66 ± 0.2^{b}	8.01 ± 0.2^{b}
	M2	10%	12.17 ± 0.2^{a}	12.33 ± 0.1^{a}	11.66 ± 0.5^{a}	12.16 ± 0.2^{a}	12.16 ± 0.2^{b}	8.01 ± 0.2^{b}
	M3	20%	11.17 ± 0.2^{ab}	8.01 ± 0.2^{d}	11.83 ± 0.2^{a}	11.66 ± 0.2^{ab}	14.16± 0.2^{a}	9.01 ± 0.2^{b}
	M4	40%	7.01 ± 0.2^{d}	7.01 ± 0.1^{e}	7.65 ± 0.3^{c}	7.01 ± 0.1^{c}	13.33 ± 0.2^{ab}	9.01 ± 0.2^{b}
**YPD + dextrose**	M5	5%	11.12 ± 0.2^{ab}	11.1 ± 0.2^{b}	10.16 ± 0.2^{b}	10.16 ± 0.5^{b}	12.51 ± 0.2^{b}	11.01 ± 0.2^{ab}
	M6	10%	11.66 ± 0.5^{ab}	11.67 ± 0.2^{ab}	10.75 ± 0.5^{ab}	10.16 ± 0.5^{b}	12.76 ± 0.2^{b}	11.01 ± 0.2^{ab}
	M7	20%	8.02 ± 0.2^{d}	7.01 ± 0.1^{d}	8.33 ± 0.5^{c}	7.01 ± 0.1^{c}	12.83 ± 0.2^{b}	8.01 ± 0.2^{b}
	M8	40%	8.02 ± 0.2^{d}	7.01 ± 0.1^{d}	7.37 ± 0.5^{c}	7.01 ± 0.1^{c}	13.33 ± 0.5^{ab}	8.01 ± 0.2^{b}
**YPD**	M9	None	7.01 ± 0.1^{d}	7.01 ± 0.1^{d}	7.01 ± 0.1^{c}	7.01 ± 0.1^{c}	7.01 ± 0.1^{c}	12.17 ± 0.2^{a}

Data are means ± standard error. Values in the same column with small letter are significantly different ($P < 0.05$). RIA (%) = 1-(Ac − As/Ac) × 100, where Ac is the inhibition zone of control sample (medium with no sugar added); As is the inhibition zone of test sample (medium with sugar). Legend: M1: M9 + 5% sucrose; M2: M9 + 10% sucrose; M3: M9 + 20% sucrose; M4: M9 + 40% sucrose; M5: M9 + 5% dextrose; M6: M9 + 10% dextrose; M7: M9 + 20% dextrose; M8: M9 + 40% dextrose. M9: YPD broth.
