# Supplementary material for: Inhibitory-like Substances Produced by Yeasts Isolated from Andean Blueberries: Prospective Food Antimicrobials
Source: Foods. 2023 Jun 21;12(13):2435. doi: 10.3390/foods12132435 (PMC10340612; doi:10.3390/foods12132435)
Supplement: Supplementary file 1 [file foods-12-02435-s001.zip › foods-2445305-supplementary-Table S3.pdf]

Table S3. Effect of enzymes on the antimicrobial activity towards *E. coli.*

Yeasts	CFS+ Enzymes (1mg/mL)					CFS	NCFS
	PK	Lys	α Chymotrypsin	Catalase	Pepsin		
Lev6	12.01 ± 0.29[a]	11.01 ± 0.29[ab]	11.83 ± 0.29[ab]	10.83 ± 0.29[b]	11.17 ± 0.29[ab]	12.83 ± 0.29[a]	7.02 ± 0.10[c]
Lev8	11.51 ± 0.29[ab]	12.17 ± 0.29[a]	11.83 ± 0.47[b]	12.33 ± 0.29[a]	11.67 ± 0.29[b]	12.33 ± 0.29[a]	7.02 ± 0.10[c]
Lev9	11.33 ± 0.28[b]	12.17 ± 0.29[a]	12.03 ± 0.47[a]	11.33 ± 0.29[b]	11.83 ± 0.29[a]	11.89 ± 0.47[a]	7.02 ± 0.10[c]
Lev15	11.12 ± 0.28[b]	12.01 ± 0.28[a]	12.01 ± 0.28[a]	11.15 ± 0.29[b]	11.51 ± 0.29[ab]	12.16 ± 0.28[a]	7.02 ± 0.10[c]
Lev30	13.66 ± 0.28[b]	14.01 ± 0.29[a]	14.01 ± 0.29[a]	13.50 ± 0.29[b]	13.17 ± 0.29[b]	14.17 ± 0.29[a]	7.02 ± 0.10[c]
SSB	11.51± 0.28[b]	12.51 ± 0.29[a]	12.66 ± 0.28[a]	11.55 ± 0.29[b]	11.67 ± 0.29[b]	12.17 ± 0.28[a]	7.02 ± 0.10[c]

Data are means ± standard error. Values in the same raw with small letter are significantly different ($P < 0.05$); NCFS (neutralized CFS heat at 80°C and pH 6.0): CFS: cell-free supernatant.
